# Transformation of Lactotroph Pituitary Adenoma to Metastatic Pituitary Carcinoma: A Case Report

**DOI:** 10.7759/cureus.95326

**Published:** 2025-10-24

**Authors:** Keegan Christensen, Midhad Mrvoljak, Chinyere L Njoku, Shrikiriti S Rajan, Moses O Evbuomwan, Mark C Smith

**Affiliations:** 1 College of Medicine, University of Iowa Carver College of Medicine, Iowa City, USA; 2 College of Osteopathic Medicine, Des Moines University College of Osteopathic Medicine, Des Moines, USA; 3 Radiation Oncology, Medserve-Lagos University Teaching Hospital Cancer Centre, Lagos, NGA; 4 Radiation Oncology, University of Iowa Health Care, Iowa City, USA

**Keywords:** lactotroph pituitary adenoma, metastatic pitnet, metastatic pituitary carcinoma, palliative radiation, pituitary adenoma, pituitary neuroendocrine tumor (pitnet), spinal metastases

## Abstract

Aggressive lactotrophic pituitary adenomas rarely transform into metastatic pituitary carcinoma. Moreover, the biological drivers and molecular characteristics that differentiate pituitary adenomas from pituitary carcinomas, or a reliable biomarker for metastatic potential, remain largely unknown. Therefore, long-term monitoring through clinical examinations, laboratory testing, and imaging is crucial for the early detection of such transformations. This approach can lead to aggressive multimodal treatments, including systemic therapies and radiation. Here, we present a rare case involving a 35-year-old male patient who developed an aggressive lactotrophic pituitary adenoma resistant to surgery, radiation, and medical treatments. Five years after undergoing adjuvant radiation therapy to the pituitary gland, the patient's condition progressed, resulting in distant spinal and bony metastases. He completed palliative radiation therapy for these metastases and underwent four cycles of combination immunotherapy for maintenance. Unfortunately, he was later diagnosed with immune checkpoint inhibitor-related hepatitis due to elevated liver enzymes. The case highlights the likely contribution of subtotal resection and high proliferative potential to incomplete remission, as patients without complete remission may have an increased risk of metastatic transformation. Thus, the need for early genetic testing and a prompt multimodal treatment approach is crucial.

## Introduction

Pituitary adenomas (PAs) are tumors that develop from hormone-secreting neuroendocrine cells in the pituitary gland. They are the third most common type of intracranial tumor after gliomas and meningiomas [1], with an estimated prevalence of approximately 10% [2]. While most PAs are asymptomatic, some can become clinically significant due to the overproduction of pituitary hormones or because they exert pressure on surrounding structures. Prolactinomas are the most common type of functional pituitary tumor, making up 44%-67% of clinically relevant neoplasms [3]. Prolactinomas exhibit symptoms that vary by sex and age due to elevated prolactin levels [4]. In children, they can cause delayed puberty. In women, symptoms include acne, menstrual disturbances (oligomenorrhea and amenorrhea), nipple discharge, and painful intercourse due to vaginal dryness [4]. In men, symptoms often involve erectile dysfunction, muscle loss, and reduced body hair [4]. Both sexes may experience decreased libido, infertility, weight gain, mood disorders, headaches, visual field defects, and hypopituitarism [4]. Overall, prolactinomas can significantly impact reproductive, neurological, and metabolic health of a patient.

Distant metastases occur in only 0.1%-0.2% of PAs, which have historically been classified as pituitary carcinomas (PCs) [5]. Because there are no distinct morphologic or molecular features to differentiate PAs from PCs, the WHO 2022 classification of pituitary tumors has redefined the entire range of pituitary tumors under the term “pituitary neuroendocrine tumor” (PitNET) [6]. PCs, or metastatic PitNETs, often do not respond well to various treatment modalities and have a poor prognosis, with an average survival time of about 10 months. Certain tumor subtypes, invasive behavior, and high Ki-67 expression levels have been linked to more aggressive tumors, but a reliable biomarker for metastatic potential has yet to be identified [7]. The rarity of metastatic PitNETs makes them challenging to study.

In this report, we describe a case of an aggressive lactotrophic pituitary adenoma that proved resistant to surgery, radiation, and medical treatment, which progressed to develop distant spinal metastases five years after adjuvant radiation therapy was administered to the pituitary gland.

## Case presentation

**Figure 1:**
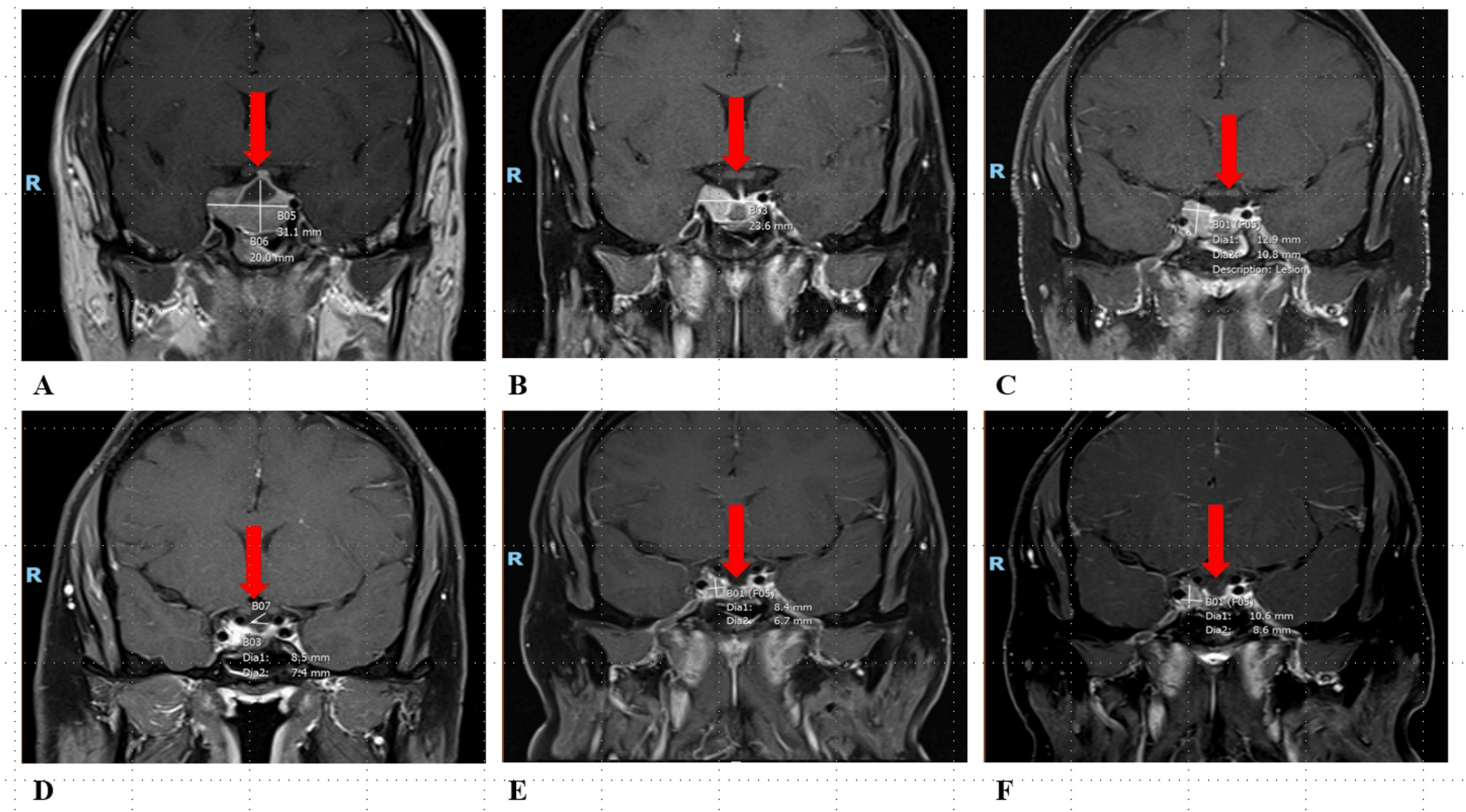
MRI coronal section T 1 post-gadolinium pre- and post-surgical intervention. A: Fall 2018: There is a T1-isointense mixed cystic and solid sellar mass with suprasellar extension. It is slightly increased in size, measuring 1.8x3.0x2.2 cm, with interval-increased cystic component. The mass extends laterally to the right and partially encases the distal right internal carotid artery. There is a mild mass effect on the optic chiasm. B: Mid-summer 2019: Stable enhancing soft tissue mass involving the sella with involvement of the cavernous sinus on the right side. Currently measuring approximately 0.9x1.7x2.4 cm in the craniocaudal, anteroposterior, and mediolateral dimensions. Stable involvement of the cavernous sinus on the right side. There is no mass effect on the optic chiasm. C: Late summer 2019: Postoperative changes of transphenoidal tumor debulking with stable enhancing soft tissue sellar mass. There is a grossly stable appearance of the residual mass measuring 1.1x1.8x2.4 cm with extension into the right cavernous sinus - unchanged mild leftward deviation of the pituitary stalk. D: Fall 2022: Stable appearance of residual pituitary adenoma. The enhancing residual lesion measures 1.9x1.1 cm in the craniocaudal and anteroposterior dimensions. The ventricular system is normal in size and configuration. There is no evidence of mass, mass-effect, hemorrhage, or extra-axial collection. E: Spring 2024: New or increased size of the right sellar/cavernous sinus enhancing lesion. New or increased size of a right sellar/cavernous sinus-enhancing lesion measuring 0.8x0.7 cm. This abuts but does not compress the right cavernous internal carotid artery - no mass effect on the optic chiasm. F: Summer 2024: Postsurgical changes of transsphenoidal pituitary mass resection. Increased size of the right sellar/cavernous sinus-enhancing mass measuring 1.1x0.9 cm in maximal coronal dimensions, with approximately 180-degree encasement of the cavernous ICA without significant vessel compression. No mass effect on the optic chiasm. Red arrow: soft tissue mass.

A 35-year-old man visited his physician in the spring of 2018 with symptoms including headaches, double vision, excessive thirst and urination, nausea, and vomiting. A magnetic resonance imaging (MRI) scan of his brain revealed a 2.7 cm mass above the pituitary gland (Figure 1A).

Baseline prolactin levels were measured and found to be elevated at 2448 ng/mL (Table 1).

**Table 1:** Patient’s laboratory panel showing prolactin level pre- and post-surgery, radiation and medical management interventions. ^Obtained after primary resection ^^Obtained after completion of radiation therapy *Outside laboratory results with a different reference reported in brackets. TSH: Thyroid-stimulating hormone.

Date	Prolactin	Testosterone Total	TSH	T4 (Thyroxine), Free	Cortisol Plasma
Reference	4.0-15.2 ng/ml	249-836 ng/dL	0.27-4.20 µIU/mL	0.8-1.8 ng/dL	6.0-18.4 µg/dL
08/01/2018	2448.0 ng/ml	<5 ng/dL	1.17 µIU/mL	0.52 ng/dL	2.7 µg/dL
12/13/2018	1373.0 ng/ml	<5 ng/dL	-	1.36 ng/dL	8.0 µg/dL
02/08/2019^	473.2 ng/ml	-	-	-	-
09/11/2019^^	221.2 ng/ml	82 ng/dL	-	1.12 ng/dL	5.0 µg/dL
12/18/2019	138.9 ng/ml	85 ng/dL	-	1.11 ng/dL	4.7 µg/dL
10/20/2021	68.6 ng/ml	6	-	0.25 ng/dL	1.7 µg/dL
07/18/2022	-	12 (123-814)* ng/dL	-	0.8 (0.8-1.5)* ng/dL	-
04/05/2023	861.0 ng/ml	<5 ng/dL	-	0.99 ng/dL	-
04/26/2024	>4700 ng/ml	<5 ng/dL	<0.01 µIU/mL	0.9 ng/dL	-

Laboratory results indicated a lactotrophic pituitary adenoma, which led to secondary adrenal insufficiency and hypothyroidism. He was started on cabergoline at a dosage of 0.5 mg twice weekly, along with thyroid and cortisol supplementation, consisting of 75 mcg of levothyroxine and 20 mg of hydrocortisone. A follow-up prolactin level checked in the fall of 2018 showed continued elevation. Additionally, a subsequent MRI at this time revealed a growing macroadenoma with cystic degeneration (Figure 1B).

In the winter of 2019, the patient underwent an endoscopic transsphenoidal pituitary tumor resection. The final pathology confirmed the presence of a lactotrophic pituitary adenoma (partially granulated) with an elevated proliferative Ki-67 rate of 22.4% positivity. Intraoperatively, the tumor was noted to be fibrous and difficult to separate from the surrounding normal tissue, preventing a gross total resection. Postoperatively, while prolactin levels improved, he continued to require cabergoline at 2 mg twice weekly.

Due to the incomplete resection and increased cabergoline requirement (3 mg twice weekly), he was evaluated for adjuvant radiation treatment in the spring of 2019. A physical examination revealed a mild saccadic ocular motion abnormality in the horizontal direction, and a review of systems identified various symptoms consistent with panhypopituitarism. He underwent external beam radiation therapy, receiving a total dose of 54 Gy in 30 sessions targeted at the residual tumor and surrounding postoperative bed, completing this treatment in the summer of 2019. Throughout the therapy, he remained on cabergoline at a dosage of 4 mg weekly. Following completion of the treatment, a brain MRI demonstrated stable disease (Figure 1C), while prolactin levels continued to decrease (Table 1).

In 2019, the patient began using gel-based topical testosterone supplementation and later tapered off hydrocortisone. By the spring of 2020, he ceased testosterone supplementation due to intolerance. In the fall of 2021, two years after completing radiation therapy, he reported increased fatigue, decreased appetite, mental fog, and approximately 20 pounds of unintentional weight loss. Although prolactin levels appeared low, he was diagnosed with adrenal insufficiency. He restarted hydrocortisone and began using gel-based testosterone again. During a follow-up visit later that year, he reported minimal compliance with testosterone due to difficulties integrating it into his daily schedule. He continued to face hormonal imbalances, necessitating increased doses of hydrocortisone and levothyroxine, while prolactin levels stabilized at 68.6 ng/mL.

In 2022, he continued to require increased doses of hydrocortisone for adrenal insufficiency, with stable prolactin levels. At this time, he transitioned to weekly testosterone injections. However, he remained medically refractory to therapy, partially due to compliance issues. An MRI from the fall of 2022 indicated increased enhancement of the tumor around the internal carotid artery (ICA) (Figure 1D). The patient was instructed to schedule close follow-up appointments.

**Figure 2:**
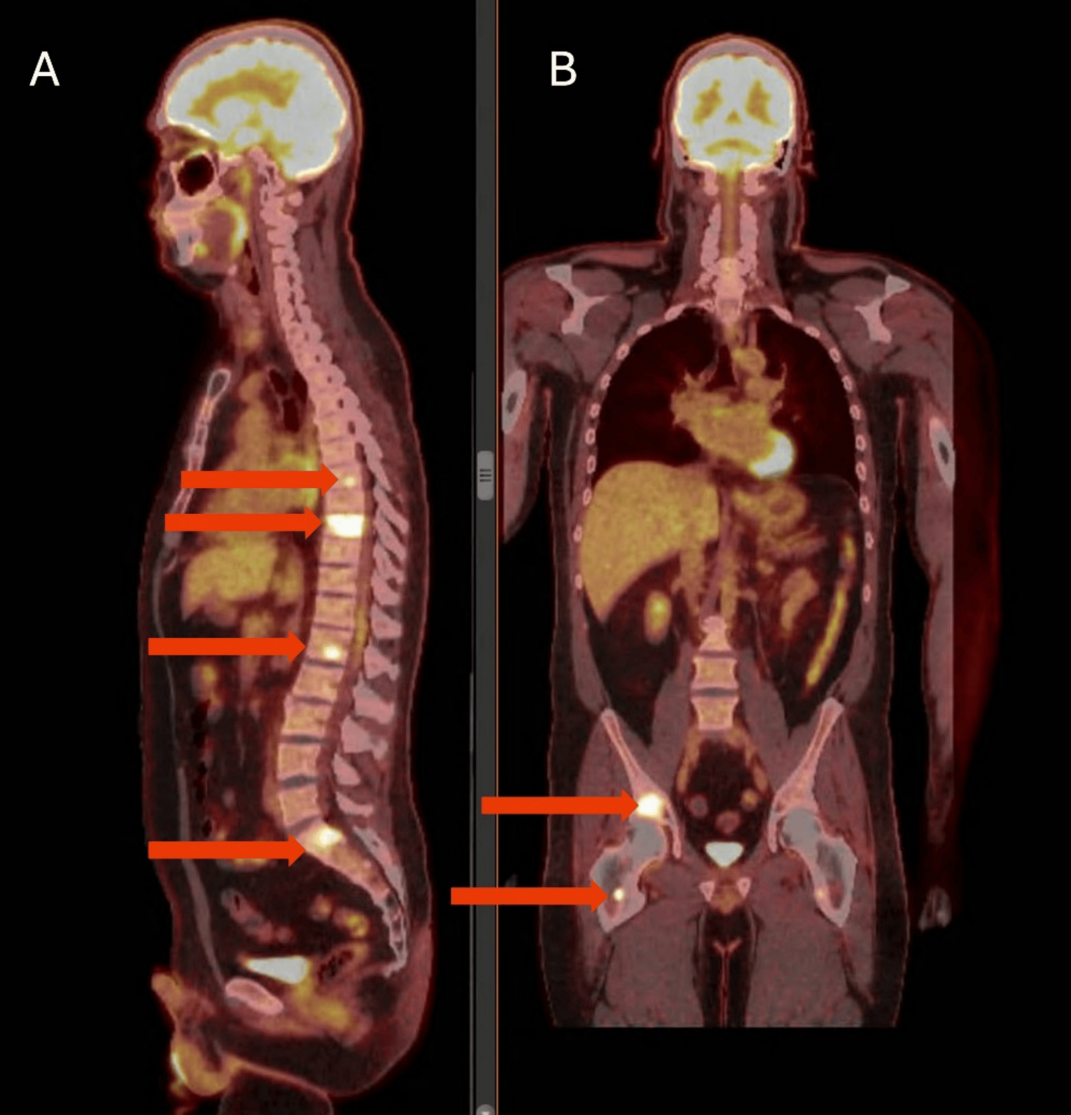
Whole-body PET/CT F-18 fluorodeoxyglucose (FDG): (A) sagittal and (B) coronal view (Summer 2024): Multiple hypermetabolic osseous lesions in the axial and appendicular skeleton concerning for metastatic disease. The red arrow shows PET FDG-avid lesion.

In the spring of 2023, he reported symptoms of blurry vision and signs of hypogonadism, including low libido and hair loss. During this visit, his prolactin level sharply elevated to 861 ng/dL. He transitioned to prednisone at 5 mg daily for convenience and symptom management, with cabergoline dosage increasing to 4 mg twice weekly. The MRI reported stable enhancing tissue. A month later, a repeat prolactin level showed stability with a mild decrease, prompting the decision to continue medical therapy and observation. He missed his scheduled six-month follow-up and returned for repeat imaging in the spring of 2024. The brain MRI demonstrated an increase in the size of the right sellar/cavernous sinus enhancing lesion, growing from 5x3 mm to 8x7 mm (Figure 1E). In the clinic, he reported new headaches and ringing in his left ear. Laboratory results showed significant abnormalities: prolactin levels > 4,7000 ng/dL, total testosterone levels below 5 ng/dL, and thyroid-stimulating hormone (TSH) levels below 0.01 µIU/mL, with normal T4 at 0.9 µIU/mL. Whole-body imaging in the spring of 2024 revealed rounded, non-enlarged lymphadenopathy in the paraesophageal and retrocrural areas (Figure 1F). Further imaging with PET/CT detected multiple hypermetabolic osseous lesions (Figure 2A, 2B); notably, the T9 vertebra displayed marked involvement (SUV max 11.6), indicating metastasis.

Two months later, he underwent a biopsy of the T9 vertebrae, revealing atypical lactotroph cells consistent with pituitary carcinoma, with negative programmed death-ligand 1 (PDL-1) testing. Gene sequencing revealed a frameshift mutation in the MEN1 gene.

The patient was advised to undergo palliative radiation therapy targeting the T6-L2 and sacral regions in July 2024. He completed 16 Gy of the planned 20 Gy. Treatment was discontinued early because of nausea that did not respond well to oral antiemetics. Given concerns about chemotherapy-induced nausea, the patient decided against chemotherapy and instead began nivolumab, ipilimumab, and zolendronic acid in the fall of 2024. Afterward, he completed palliative radiation treatment, receiving 20 Gy to the bilateral femurs, without any complications. He underwent four cycles of combined immunotherapy but was presumptively diagnosed with immune checkpoint inhibitor-related hepatitis due to elevated liver enzymes. He was treated with a steroid (methylprednisolone 100 mg IV daily switched to prednisone 100 mg daily), which resulted in a downtrend in liver enzymes (Table 2).

**Table 2:** Patient's liver function laboratory panel showed values at the start of cycle 1 combination immunotherapy on September 6, 2024 and cycle 4 combination immunotherapy on November 22, 2024. The patient was started on steroids on December 15, 2024 because of the elevated values. ^* Outside laboratory results from December 15, 2024 to January 17, 2025 with a different reference reported in brackets.

Date	Albumin	ALP (Alkaline Phosphatase)	ALT (Alanine Aminotransferase)	AST (Aspartate Aminotransferase)	Bilirubin Total	Total Protein
Reference	3.4-4.8 g/dL	40-129 U/L	0-41 U/L	0-40 U/L	≤1.2 mg/dL	6.0-8.0 g/dL
09/06/2024	4.3 g/dL	137 U/L	47 U/L	38 U/L	0.2 mg/dL	7.3 g/dL
11/22/2024	4.0 g/dL	136 U/L	59 U/L	33 U/L	0.1 mg/dL	6.8 g/dL
12/15/2024*	3.4 g/dL	408 (40-150) U/L	3533 (<56) U/L	4078 (5-34) U/L	4.8 (0.2-1.2) mg/dL	6.7 (6.4-8.3) g/dL
12/17/2024	3.1 g/dL	364 U/L	2212 U/L	1113 U/L	3.5 mg/dL	5.9 g/dL
01/17/2025	3.6 g/dL	211 U/L	825 U/L	224 U/L	1.6 mg/dL	6.1 g/dL

As a result, the medical team decided not to continue with maintenance nivolumab. He also declined plans to undergo repeat PET/CT and MRI of the brain to evaluate the tumor response to his four cycles of combination immunotherapy.

## Discussion

Pituitary macroadenoma, a condition that is often benign, can exhibit local invasiveness in about 40% of cases. Metastatic PitNET, though exceedingly rare, is reported in approximately 0.1%-0.4% of all cases [8-10]. According to the WHO 2022 report, no single clinical, radiological, or histological parameter can reliably predict the risk of growth or metastatic potential [8]. The study of these tumors is crucial, as the median overall survival following metastasis is only 1.5 years [10]. In this report, we present a rare case of metastatic PitNET and explore potential markers for its metastatic potential.

For lactotrophic pituitary macroadenomas, the first-line treatment is often cabergoline, a dopamine agonist [11-13]. Most patients respond to this treatment within six months. In our review of the current literature indexed in PubMed, fewer than 30 cases of metastatic lactotrophic pituitary carcinoma have been documented. Most reports indicate that primary therapy typically involves a dopamine agonist such as cabergoline or bromocriptine, followed by surgery. Our patient did not achieve remission with cabergoline alone and underwent surgery within two years of diagnosis, which is consistent with existing literature [10]. The interval to metastasis without complete remission is frequently cited as occurring within 10 years of the initial diagnosis and completion of therapy, with times to metastatic progression ranging from 0 to 43 years [14].

For patients who are medically refractory, secondary therapies may include external beam radiation therapy, occasionally alongside stereotactic radiosurgery [11,12]. Although the use of radiotherapy in treating pituitary adenomas is relatively common, with a local control rate exceeding 90%, the treatment plans and optimal doses used for metastatic lactotrophic pituitary carcinoma are not uniform across cases. While increased metastatic potential has been reported in brain tumors previously treated with radiotherapy, this cannot be definitively linked to carcinoma conversion and metastases in our patient [12]. While secondary malignancies following radiotherapy to brain parenchyma can occur, most common reports are of secondary glioma or meningioma [15], particularly in following radiotherapy treatment to pediatric brain tumors and pituitary adenomas. In contrast, cases of metastatic transformation are often primarily linked to aggressive histological features, rather than the use of radiotherapy, such as adrenocorticotropic hormone (ACTH) secretion or high Ki-67 [8]. It is possible that radiotherapy may play a more direct role, as one such case study identified somatic mutations following radiotherapy that appear to be deleterious in protein function [16]. However, it is unclear how pivotal these mutational changes may have been in the transformation to metastatic carcinoma within a few years after radiation treatment. Furthermore, despite these concerns, radiotherapy remains a mainstay in treatment algorithms for aggressive secretory pituitary tumors [8] with the goal of providing local and biochemical control.

In this case, the patient's medical challenges and systemic side effects led to his decision not to pursue further imaging to evaluate the tumor's response after four cycles of combination immunotherapy. If the scans were to reveal a non-osseous site of metastasis, a biopsy could be considered to test for O6-methylguanine-DNA methyltransferase (MGMT) methylation status. This information would help determine the likelihood of the tumor's susceptibility to temozolomide. According to the European Society of Endocrinology's 2016 survey, of the 157 patients with aggressive PitNET and carcinoma treated with temozolomide, 37% exhibited a radiological response, and 19% demonstrated a complete biochemical response [17]. Clinically functioning tumors, low MGMT, and concurrent radiotherapy were associated with a better response [17]. Ultimately, in our case, the decision to pursue immunotherapy over targeted agents was fueled by the patient’s personal desire to maintain quality of life and approach minimally toxic therapies. Other limitations also include metastatic disease limited to osseous sites, rather than soft tissue sites not including brain that would have lent better to comprehensive testing with increased diagnostic yield.

In the WHO 2022 update concerning aggressive and metastatic PitNETs, certain characteristics - such as macroadenomas, corticotrophic presentation, increased mitotic activity, and high Ki-67 indices - were noted to suggest aggressive behavior, though not directly linked to metastatic potential [8]. Our patient presented with significant mass effect, intraoperative adherence, unusually fibrous characteristics, and a high Ki-67 index of 22.7%. These features have been discussed in several other studies [13] and indicate a potential area for classification modifications to better identify tumors with metastatic potential, which would allow for more aggressive initial management strategies involving surgery and adjuvant radiation therapy.

Lastly, studies have reported various somatic gene mutations associated with aggressive behavior and metastatic dissemination [8,9,18,19], primarily in corticotrophic pituitary tumors. Evidence of such mutations in lactotrophic pituitary adenomas is much scarcer. In our patient, we identified a somatic MEN1 frameshift mutation that occurred during the metastatic transformation. This is distinct from the MEN1 germline mutation associated with multiple endocrine neoplasia syndrome. MEN1 has previously been identified as a tumor suppressor gene with key roles in cell proliferation. Somatic mutations in MEN1 have been identified in other tumor types but have yet to be shown directly related to carcinoma conversion or to metastatic transformation [8]. Gene sequencing repertoires from studies conducted at the time of initial surgery are rare in patients due to the general low metastatic potential of this tumor. However, as the utilization of such studies increases, it may enhance our ability to identify markers of metastatic potential early and discover specific molecular targets for treatment.

## Conclusions

Metastatic pituitary neuroendocrine tumors (PitNET) are quite rare, accounting for only 0.4% of all PitNET cases. In this report, we describe a case that began as a pituitary adenoma, which had a relatively prolonged period without achieving complete remission. In this report, we have showed that subtotal resection and high Ki-67 positivity are likely contributing factors to the incomplete remission observed. The role of radiotherapy in providing local and biochemical control is key, but its potential contribution to carcinoma conversion or metastatic transformation remains unclear. However, given the short time interval between post-radiation therapy and the rise in prolactin levels, this likelihood appears to be low. Although somatic mutations in MEN1 have not been directly linked to carcinoma conversion or metastatic transformation, we recommend early genetic testing for potential metastatic markers associated with these somatic mutations. This is important because patients without complete remission may have an increased risk of metastatic transformation. Given the rare and highly debilitating nature of these tumors, ongoing research and prompt interventions are crucial for improving patient outcomes.
